# Improved Rubber Performance Through Phenolic Resin-Modified Silica: A Novel Coupling Mechanism for Enhanced Recyclability

**DOI:** 10.3390/polym17111437

**Published:** 2025-05-22

**Authors:** Pilar Bernal-Ortega, Rafal Anyszka, Raffaele di Ronza, Claudia Aurisicchio, Anke Blume

**Affiliations:** 1 Department of Mechanics of Solids, Surfaces & Systems (MS3), Faculty of Engineering Technology, University of Twente, 7522 NB Enschede, The Netherlands; r.p.anyszka@utwente.nl (R.A.); a.blume@utwente.nl (A.B.); 2 Bridgestone EU NV/SA, Italian Branch—Technical Center, Via del Fosso del Salceto, 00128 Rome, Italyclaudia.aurisicchio@bridgestone.eu (C.A.)

**Keywords:** silica, tires, silane, sustainability, coupling, recyclability, phenolic resin

## Abstract

Passenger car tires (PCTs) usually consist of a silica/silane-filled Butadiene Rubber (BR) or Solution Styrene Butadiene (SSBR) tread compound. This system is widely used due to improvements observed in rolling resistance (RR) as well as wet grip compared to carbon black-filled compounds. However, the covalent bond that couples silica via silane with the rubber increases the challenge of recycling these products. Furthermore, this strong covalent bond is unable to reform once it is broken, leading to a deterioration in tire properties. This work aims to improve these negative aspects of silica-filled compounds by developing a novel coupling system based on non-covalent interactions, which exhibit a reversible feature. The formation of this new coupling was accomplished by reacting silica with silane and a phenolic resin in order to obtain simultaneous π–π interactions and hydrogen bonding. The reaction was performed using two different silanes (amino and epoxy silane) and an alkyl phenol–formaldehyde resin. The implementation of the new coupling resulted in improved crosslink density, better mechanical performance, superior fatigue behavior, and a similar rolling resistance indicator.

## 1. Introduction

One of the main challenges to achieve sustainability in the rubber industry is reducing waste generation and improving the existing recycling methods. Managing and treating rubber waste, particularly from End-of-Life Tires (ELTs), is a major challenge [1,2,3]. Tires have a complex composition and structure. For example, the tread of a Passenger Car Tire (PCT) consists of a mixture of natural and synthetic rubbers, silica, silane, carbon black, oils and chemical additives. Additionally, the chemical crosslinks between the polymer chain as well as between silica and the polymer via silane, which are formed during the vulcanization process, make rubber recycling even more difficult [2,4,5].

Nowadays, a large number of tires is recycled, especially in Europe [2]. The main methodologies used for recycling of tires are mechanical recycling, pyrolysis, and devulcanization [3,6,7,8]. The ground rubber obtained after the mechanical recycling process can be used in low-value applications such as asphalt or playground surfaces [2]. In the case of pyrolysis, useful products like recovered carbon black or oil are obtained that can be used again in the production of tires. However, the pyrolysis process consumes high amounts of energy and the recovered carbon blacks obtained have a low reinforcing potential [9]. Lastly, the devulcanization of rubber aims to break down the chemical sulfur crosslinks formed during the vulcanization process and go back to the original state of the material to be able to use it again in the production of new tires [6,10]. Still, devulcanization is a complex process, in which only di- and polysulfidic bonds can be broken. The obtained materials show a high deterioration of the in-rubber properties compared to the virgin rubber, which limits its application.

Today, modern passenger car tire tread compounds consist of a silica–SBR-reinforced system. This system is widely used due to the improvements observed in the rolling resistance compared to carbon black-filled compounds [11,12,13]. Nevertheless, the polar silica particles do not have a good interaction with the non-polar rubber. To improve the compatibility of silica with rubber, silane coupling agents are needed. Silanes are bi-functional structures that act as “bridges” between the silica surface and the rubber [14,15,16]. They create a chemical bond with the filler and the elastomer, enhancing in this way the in-rubber properties of silica-filled compounds. However, the presence of these covalent bonds is an additional challenge when recycling tire waste. Another drawback of the silica–rubber coupling is that it is short and stiff, and once it is broken under strain, it is not able to re-connect [17,18]. An additional disadvantage in the devulcanization of silica-filled systems is the nature of the silica−silane−polymer bonds. These systems are primarily composed of C−S−C monosulfidic bonds, which are highly stable and resistant to cleavage. As a result, selective breaking the polymer−sulfur−polymer bonds becomes nearly impossible. Consequently, the devulcanization process yields low-quality outcomes.

In recent years, one of the more studied approaches is the implementation of reversible bonds on rubber materials instead of strong covalent bonds to facilitate the devulcanization process [18,19,20,21,22,23,24]. In this regard, non-covalent interactions have been widely studied. Reversible non-covalent interactions are weaker than covalent bonds, but they show the ability to be (re-)formed and broken under certain conditions or stimuli, such as pressure, temperature, or the application of a magnetic or electric field [21,25]. This characteristic makes these bonds extremely attractive for polymeric materials in order to achieve products with higher durability or self-healing properties. Some examples of the most studied non-covalent interactions in rubber compounds are hydrogen bonding, π–π stacking, or cation–π interactions [19,20,26,27,28].

This study presents the development of a novel silica–rubber coupling based on non-covalent interactions with the aim of providing self-healing properties and facilitate the recycling of silica-filled compounds. This new coupling, based on π–π stacking interactions, was achieved through the use of a phenolic resin and silane. Phenolic resins are synthetic polymers derived from the reaction between phenol and formaldehyde [29,30,31]. They are known for their high thermal stability, mechanical strength, and chemical resistance [29,30,32]. In rubber compounds, they are mainly used as reinforcing, tackifying, or bonding agents. The addition of phenolic resins in rubber compounds leads to improvement in properties such as heat resistance, mechanical strength, and adhesion with other materials [33,34,35,36]. The aromatic rings present in the chemical structure of the phenolic resin might interact with the styrene units of the styrene–butadiene rubber through π–π stacking. Furthermore, if the resin has good compatibility with the SBR, physical interactions between the resin and the rubber might occur.

In this work, the formation of this new coupling was accomplished by the reaction of silica with silane and afterwards with a phenolic resin in order to obtain simultaneous π–π interactions and hydrogen bonding. The reaction was performed using two different silanes, 3-Aminopropyltriethoxysilane and 3-Glycidyloxypropyltrimethoxysilane. Additionally, an alkyl phenol–formaldehyde resin was used. The in-rubber results obtained for this novel coupling were compared with a traditional silica–silane-rubber coupling using bis(triethoxysilylpropyl) disulfide as the coupling agent.

## 2. Materials and Methods

### 2.1. Materials

The rubber compounds analyzed in this work were prepared using Solution Styrene Butadiene Rubber (SSBR) and Butadiene Rubber (BR) as the polymers and silica as the filler. The polymers used in this work were Sprintan 4601 (SSBR, Synthos, Schkopau, Germany) and Buna CB24 (BR, Arlanxeo, Dormagen, Germany). Silica employed is a highly dispersible and reinforcing precipitated silica, ULTRASIL 7000 GR (Evonik Industries, Wesseling, Germany) with a specific BET surface area of approximately 175 m^2^/g. For the preparation of the reference samples, Bis(triethoxysilylpropyl) disulfide silane (TESPD) (Evonik Industries, Wesseling, Germany) was used as a coupling agent. For the in situ modification of silica, 3-Aminopropyltriethoxysilane (Sigma Aldrich, Zwijndrecht, The Netherlands) and 3-Glycidyloxypropyltrimethoxysilane (Sigma Aldrich, Zwijndrecht, The Netherlands) were used as silanes and an alkyl phenol–formaldehyde resin (DUREZ 19900, Sumitomo Bakelite Europe, Ghent, Belgium) as the resin. The rest of ingredients used in the rubber formulations were as follows: zinc oxide (ZnO) and stearic acid were used as activators (Millipore Sigma, Hamburg, Germany); sulfur and N-tert-butyl-benzothiazole sulfenamide (TBBS) (Caldic B.V., Rotterdam, The Netherlands) as curatives, and Treated Distillate Aromatic Extracted (TDAE) (Hansen and Rosenthal, Hamburg, Germany) as oil.

### 2.2. Compounding and Mixing

The samples studied in this work were prepared according to the formulation shown in Table 1. The compounds studied were as follows:

- Reference: SSBR/silica compound using TESPD as a silane coupling agent;
- AMR5: SSBR/silica compound using 3-Aminopropyltriethoxysilane as silane and 5% of the phenolic resin;
- AMR10: SSBR/silica compound using 3-Aminopropyltriethoxysilane as silane and 10% of the phenolic resin;
- EPR5: SSBR/silica compound using 3-Glycidyloxypropyltrimethoxysilane as silane and 5% of the phenolic resin;
- EPR10: SSBR/silica compound using 3-Glycidyloxypropyltrimethoxysilane as silane and 10% of the phenolic resin.

**Table 1:** Formulation of the rubber compounds.

Compounds	SBR (phr)	BR (phr)	Silica (phr)	TESPD (phr)	Amino Silane (phr)	Epoxy Silane (phr)	Resin (phr)
Reference	80	20	80	6.2	-	-	-
AMR5	80	20	80	-	5.78	-	4
AMR10	80	20	80	-	5.78	-	8
EPR5	80	20	80	-	-	6.2	4
EPR10	80	20	80	-	-	6.2	8

The amount of resin added to the samples was 5% or 10% of the amount of silica in the compounds. The amount of silane was adjusted equimolarly for each compound based on the amount of TESPD in the reference sample.

The quantities of used ingredients were the same for all compounds: TDAE—37.5 phr, ZnO—2.5 phr, stearic acid—2.5 phr, sulfur—1.4 phr, and TBBS—2 phr.

The rubber compounds were mixed in an internal mixer (Brabender Plasticorder 350S, Duisburg, Germany) with a fill factor of 0.7, an initial temperature of 100 °C for steps 1 and 2, and an initial temperature of 50 °C for step 3, at an initial rotor speed of 50 rpm. The complete mixing procedure is shown in Table 2.

### 2.3. In-Rubber Tests

The following in-rubber characterizations were performed for all rubber compounds.

#### 2.3.1. Cure Behavior

The vulcanization properties of rubber compounds were analyzed by using a Rubber Process Analyzer, RPA elite from TA instruments (New Castle, DE, USA), at 160 °C, by applying a deformation of 6.98% at a frequency of 1.667 Hz. The t_90_, the time to reach 90% conversion of each compound was used as a molding time for vulcanizing the rubber samples in a hydraulic press (Wickert Maschinenbau GmbH, Landau, Germany).

#### 2.3.2. Payne Effect

The micro-dispersion of the filler and the degree of filler–rubber interactions were analyzed by the study of the Payne effect. The Payne effect test was performed on a Rubber Process Analyzer, RPA elite from TA instruments (New Castle, DE, USA), with strain sweeps from 0.1% to 100% for cured samples at a frequency of 1.6 Hz and a temperature of 60 °C. The cured samples were vulcanized beforehand inside the equipment chamber according to the vulcanization conditions. The Payne effect is the difference between the storage moduli at 0.56% and 100% of strain.

#### 2.3.3. Macro-Dispersion of the Filler

The macro-distribution of the silica particles in the rubber compounds was studied by the analysis of the macro-dispersion of the filler. The macro-dispersion was studied by means of a Dispergrader, Dispersion Tester Alpha View (Alpha Technologies, Hudson, OH, USA). The macro-dispersion of a filler describes the degree of a filler distribution at a scale of 2 µm up to 100 µm. The rubber samples were investigated by optical light microscopy with a 30° irradiation angle and at 100× magnification.

#### 2.3.4. Crosslink Density

The crosslink density (CLD) of the compounds was studied using two different methods: equilibrium swelling experiments and stress–strain experiments using the Mooney–Rivlin approach.

- The crosslink density by equilibrium swelling experiments was obtained using the Flory Rehner equation [37]. Five vulcanized samples (~0.25 g) of each compound were swollen in toluene at room temperature for a period of 7 days. The samples were extracted before with acetone for 24 h in order to remove the oil and unreacted curing agents.
- The determination of the crosslink density by the Mooney–Rivlin approach was carried out by stress–strain assays performed in a universal testing machine Zwick Z010 (Zwick, Ulm, Germany). Test specimens were 2 mm thick, with a test length of 20 ± 0.5 mm and a width of the narrow portion of 4 ± 0.1 mm, according to the ISO 37 (dye type 2) standard [38]. The stress–strain assays consisted of two different steps:
  (i) In the first step, the samples were pre-cycled 10 times until 200% strain at a crosshead speed of 500 mm/min. This first step was performed in order to destroy the filler–filler network.
  (ii) In the second step, the specimens were stretched until a strain of 200% at a crosshead speed of 10 mm/min.

#### 2.3.5. Stress–Strain Behavior

The mechanical properties of the compounds at room temperature were measured by a universal testing machine Zwick Z05 (Zwick, Ulm, Germany) operating with a crosshead speed of 500 mm/min according to the ASTM D412 standard [39]. For each compound, five samples were tested, and the average result of these samples was determined.

#### 2.3.6. Dynamical Properties

Dynamic mechanical measurements of the vulcanized samples were carried out on a Gabo-Netzsch Eplexor tester (Netzsch, Selb, Germany). The measurements were performed with a frequency of 10 Hz, with a dynamic strain of 1% below 0 °C and 3% above 0 °C. The change in the strain was chosen because the rubber becomes softer at higher temperatures, which can generate noise in the measurements. The temperature range of the test was from −80 to 80 °C. The loss factor (tan δ) of each compound was evaluated at low (0 °C) and at high temperatures (60 °C).

#### 2.3.7. Re-Connectivity of Bonds

After the study of how the new coupling systems affect the in-rubber properties, the best compound was selected and compared with the reference. These two compounds were tested to quantify the degree of the re-connectivity of the non-covalent interactions.

The re-connectivity of the new bonds created with the silica modification was analyzed by:

- The study of the mechanical response of the compounds at high temperatures

The mechanical properties of the compounds were measured by a universal testing machine Zwick Z010 (Zwick, Ulm, Germany) operating with a crosshead speed of 500 mm/min and with a limit strain of 330%, which is the thermal chamber limit. The tests were performed in a temperature chamber at 100 °C.

- Analysis of the changes in the dynamical properties after submitting the compounds to cycling (fatigue) test

The dynamic mechanical properties of the samples were compared before and after the cyclic strain at 100 °C. The measurements of the dynamic properties of the vulcanized compounds before and after tensile cycling were performed in the same way as the dynamic mechanical measurements explained above. The tensile cycling of the samples was performed in a universal testing machine Zwick Z010 (Zwick, Ulm, Germany) operating with a crosshead speed of 500 mm/min. All the samples were cyclically strained from 0% to 100% five times at 100 °C. Immediately after performing the cycling, the dynamical mechanical properties were tested.

- Creep experiments

The creep experiments were measured by a universal testing machine Zwick Z010 (Zwick, Ulm, Germany). A force of 15 N was applied to the samples. This force was maintained for 1 h. After this time, the force was released and the changes in the strain of the samples were measured for another hour. The temperature of the experiments was 100 °C.

- Recycling test

The recyclability of the materials was tested by the study of the re-use of ground material in new compounds. The samples were cryo-ground, added to new compounds, and characterized. The samples were cryogenically ground in a grinder from Fritsch GmbH, Idar-Oberstein, Germany. The granules were frozen with liquid nitrogen and ground with a 0.6 mm sieve. This step was performed in order to facilitate the incorporation into the rubber. The properties of the compounds containing ground materials were compared to the ones of the original samples. For the preparation of the new compounds, the formulation shown in Table 1 was used, substituting 15 phr of the filler with the ground material. The ground material obtained from the selected best-performing sample was introduced to the same virgin compound, and the ground material from the reference was added to the virgin reference compound.

## 3. Results

**Figure 1:**
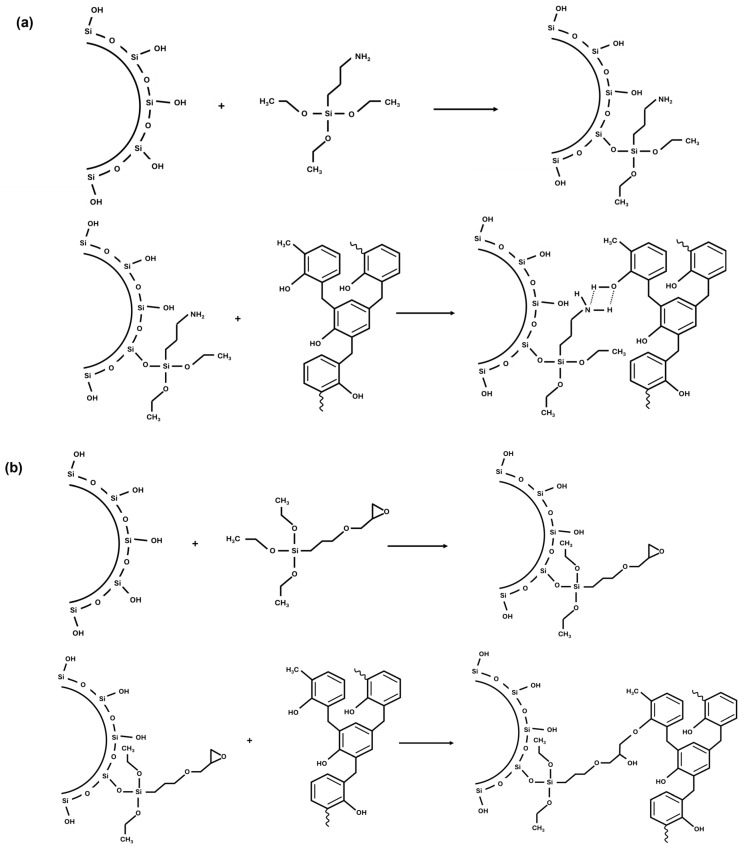

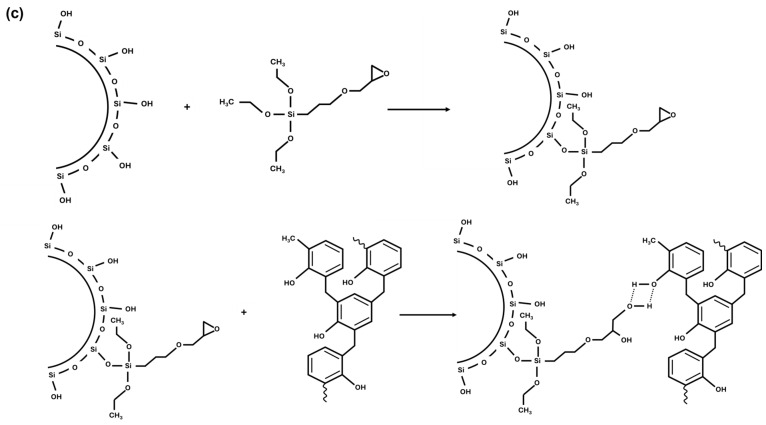
Schematic representation of the silica surface modification mechanism with the phenol resin using (**a**) amino silane, (**b**), and (**c**) epoxy silane.

The samples with the new coupling were characterized by different methodologies and compared to a reference compound with a traditional silica/silane coupling using TESPD as the coupling agent. The in situ modification of silica was performed in all cases during the mixing process. The new coupling system was developed by reacting silica with silane, followed by the reaction with a phenolic resin, to establish π–π interactions between the resin and the styrene–butadiene rubber. Two different silanes were studied, 3-Aminopropyltriethoxysilane and 3-Glycidyloxypropyltrimethoxysilane. A schematic representation of the possible reactions that can occur in the system is shown in Figure 1. In Figure 1a, the reaction using 3-Aminopropyltriethoxysilane is shown. First, the amino silane reacts covalently with the hydroxyl groups of the silica surface, and subsequently, the amino group (-NH_2_) of silane can form hydrogen bonds with the hydroxyl groups of the phenolic resin. However, in extreme conditions (e.g., extremely high temperatures, catalysis), covalent bonding may also occur; however, under the conditions used in this study, hydrogen bonding is assumed to be the dominant interaction in this system. 3-Glycidyloxypropyltrimethoxysilane (Figure 1b), as amino silane, first reacts covalently with the silica surface. Afterwards, silane can form a covalent bond with the phenolic resin via the epoxide ring-opening reaction caused by the hydroxyl groups of the resin. In addition to covalent bonding, hydrogen bonding could occur between the hydroxyl groups on the phenolic resin and the newly formed secondary hydroxyl groups on the silane after the opening of the epoxide ring (Figure 1c). During the mixing process, an additional side reaction could occur. While mixing, silane coupling agents are exposed to atmospheric moisture, leading to their hydrolysis and subsequent condensation to form siloxane networks within the rubber matrix. This reaction pathway is particularly relevant in the presence of amino silanes, which can catalyze these processes. Under such conditions, mechanisms similar to the Stöber process [40]—originally described for the controlled synthesis of monodisperse silica particles from alkoxysilanes in alcoholic media—may occur. In the compounding process, the presence of moisture and amine groups can enhance the in situ formation of silica-like domains via hydrolysis and polycondensation of silanes. This phenomenon could influence the filler dispersion and therefore the mechanical and dynamic properties of the final compound.

### 3.1. Vulcanization

**Figure 2:**
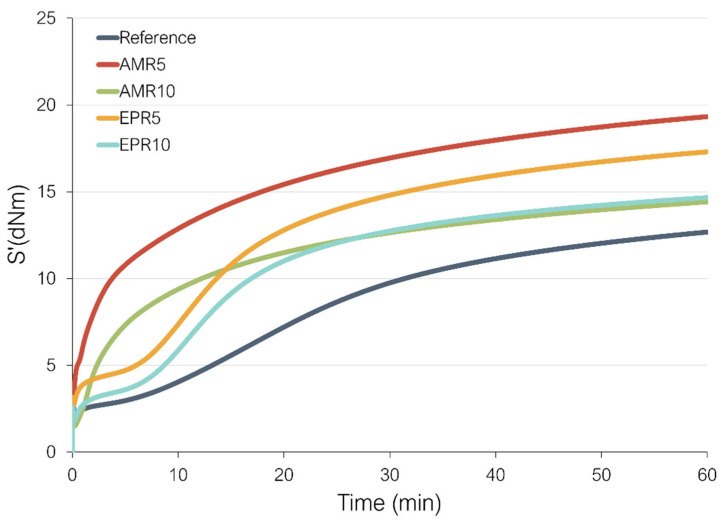
Vulcanization curves of the studied compounds.

Figure 2 shows the cure behavior of the studied samples. In the graph, it can be observed that samples containing amino silane (AMR5 and AMR10) show a faster vulcanization compared to the samples with epoxy silane and the reference. This is caused by the presence of the amino group (-NH_2_). It has been demonstrated that the amino group accelerates vulcanization because it is a base and promotes the opening of the sulfur ring and its conversion to more reactive forms [41,42]. The slow curing behavior of the reference compound is caused by the presence of only one accelerator in this sample. The compound was designed in this way to reduce the complexity of the system to avoid interference with the newly developed coupling systems. Comparing AMR5 and AMR10, the compound with only 5% resin has a faster vulcanization rate. This might be caused by a dilution effect—the higher the content of the resin, the lower the amount of elastomer per volume. Additionally, the phenolic resin can interact with the accelerator, deactivating it for the vulcanization process. The same occurs in the compounds with epoxy silane, where EPR5 undergoes faster vulcanization than EPR10. In the samples with amino silane, two opposite reactions occur in the system: the fastening of the crosslinking reaction due to the presence of the amino group and the deceleration of it due to the phenolic resin. In sample AM5, the effect of the amino silane is more predominant, but in sample AM10, with the increase in the phenolic resin content, the second reaction becomes more significant.

Figure 2 also shows that the samples with 5% of resin (AMR5 and EPR5), independently of silane, show the higher values of the maximum torque (M_H_), with AMR5 reaching the highest value in all samples. The addition of 10% of resin in both systems leads to lower M_H_. Compared to the reference, all samples with the new couplings show higher values of M_H_. Commonly a higher value of maximum torque is an indication of higher crosslink density (CLD). Therefore, these results might suggest that the samples containing resins show a higher CLD than the reference. However, a higher maximum torque can also be caused by other factors. It can also be associated with higher filler–filler interactions and thus worse dispersion in these compounds. This increase in the filler–filler interactions might be a consequence of the presence of many different interactions in these systems: filler–filler interactions (e.g., via silica–resin–silica) or polymer–filler interactions (via silica–resin–polymer).

### 3.2. Payne Effect

**Figure 3:**
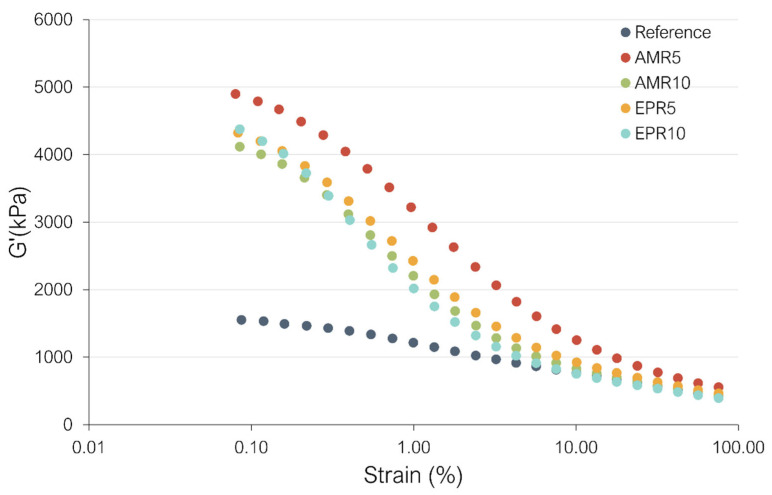
Payne effect curves of the studied compounds.

Figure 3 shows the reduction in the storage modulus (G′) with an increasing strain. This phenomenon is associated with the filler network breakdown, described in the literature as the Payne effect [43,44,45]. The Payne effect is an indirect method of analyzing the micro-dispersion of the filler in the rubber matrix [43,44,45]. A higher Payne effect, calculated as the difference between G′ at high and low strains, generally suggests a worse micro-dispersion. The Payne effect also contributes to the energy dissipation in rubber compounds, which is a key parameter in the performance of tires (wet grip and rolling resistance).

**Figure 4:**
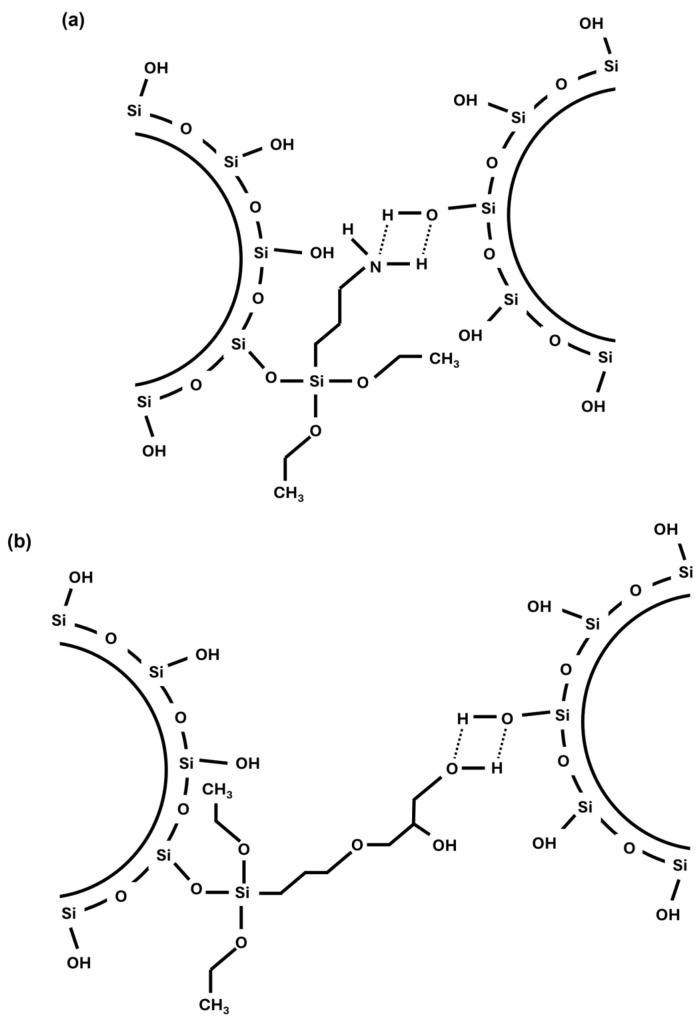
Schematic representation of the interactions between silica particles connected by (**a**) amino and (**b**) epoxy silane.

Figure 3 and Table 3 display that the reference sample has the lowest Payne effect of all samples. The compounds with the grafted resin, either with amino or epoxy silane, present significantly higher values. The sample AMR5 shows the highest Payne effect of all compounds. These results are in agreement with the higher maximum torques observed for these samples in the analysis of the cure behavior. Indicating that the higher torques observed are most likely to be caused by higher filler–filler interactions in these compounds instead of a higher CLD. Figure 3 also shows that in the case of the samples containing resin, the decay of G’ occurs faster than in the reference compound. Due to the increased polarity of the system in the compounds with the new coupling, the interaction possibilities between silica particles have significantly increased. It is possible that silanes (both amino and epoxy silane) can connect different silica particles. Both silanes can chemically couple to silica via the ethoxy group but can also form a hydrogen bond to another Si-OH from another silica particle. The distance between these silica particles might be bigger and more flexible than the one of a direct particle–particle interaction. A schematic representation of these interactions is shown in Figure 4.

Another possible explanation for the higher Payne effect in the samples with the new coupling is that not only filler–filler interactions are breaking with the applied strain. It is possible that the non-covalent interactions present in these systems, such as hydrogen bonding or π–π stacking break when strain is applied due to its weaker nature compared to covalent bonding. Therefore, in these cases, the measurement of the Payne effect is the combination of two different effects, the filler–filler and the filler–rubber interactions [18]. This effect is also known from previous studies of functionalized polymers [46], where the interaction of the functionalization is based on hydrogen bonding and the Payne effect curves show similar behavior. At low strains, the non-covalent links remain intact, contributing to a higher value of G′ as can be seen in Figure 3. As the strain increases, these weaker interactions start to break down, causing a sharp drop in G’.

Comparing the effect of the different silanes (amino and epoxy silane) it is observed that with 5% of resin, the sample with amino silane shows a substantially higher Payne effect than the one with epoxy silane. In the case of the epoxy silane, it has more direct reactivity towards the phenolic resin through the ring opening. This reaction can lead to the formation of some covalent bonds, stronger and more stable. The amino silane primarily interacts with the resin via hydrogen bonding, and therefore the interactions are weaker and more prone to be broken when deformed.

As can be seen in Table 3 and Figure 3, all samples show similar values of G′ at high strains. This parameter is associated with higher rubber–filler interactions. Therefore, this result indicates that the new compounds present similar polymer–filler interactions than the reference that could result in a comparable mechanical performance. This also indicates that not all non-covalent interactions are broken down when the strain increases. It also could be an indication that some of the non-covalent interactions are reformed.

### 3.3. Macro-Dispersion of the Filler

**Figure 5:**
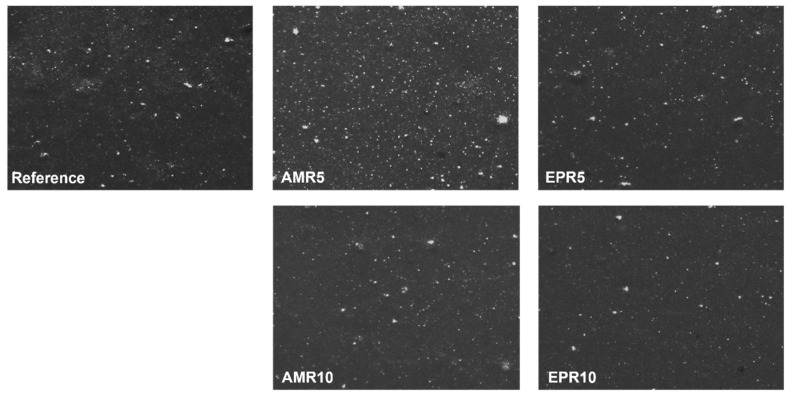
Dispergrader images of the analyzed rubber compounds (150 × 250 µm area).

The macro-dispersion of the filler in the analyzed compounds was evaluated using the optical Dispergrader (Alpha Technologies, Hudson, OH, USA) device. The resulting images are presented in Figure 5. All samples, except for AMR5, show a similar macro-dispersion of the filler. Sample AMR5 presents slightly poorer macro-dispersion, with the presence of larger clusters being noticeable. The implementation of the new coupling system and all the new possible interactions in the system (Figure 4) does not seem to have a significant effect on the macro-dispersion of the silica particles, contrary to what was observed in the Payne effect study which delivers hints on the micro-dispersion. However, both measurements refer to two different length scales and are two independent processes [43,44,45].

### 3.4. Crosslink Density

**Figure 6:**
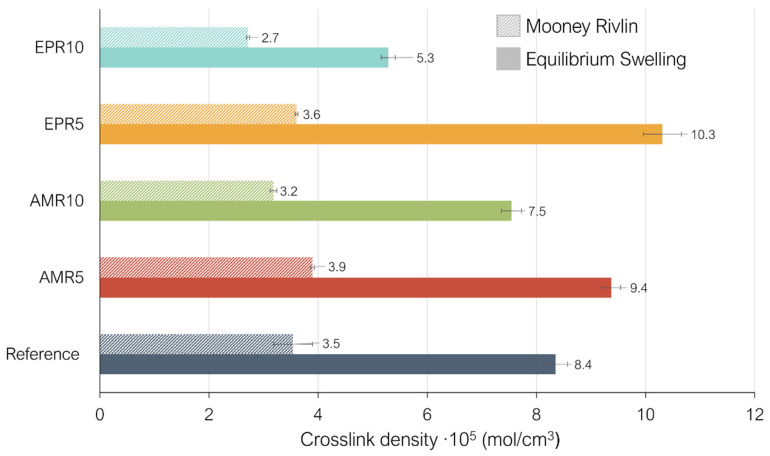
Crosslink density measured by equilibrium swelling and the Mooney–Rivlin approach applied to the studied compounds.

The crosslink density of the studied compounds is shown In Figure 6. The results obtained with equilibrium swelling correspond to the solid color bars and the ones obtained with the Mooney–Rivlin approach to the bars with stripes.

As can be seen in Figure 6, the results of both techniques show some differences. This might be caused by the differences between the two methods such as the use of a solvent in the equilibrium swelling experiments or the pre-treatment to destroy the filler network of the samples in the Mooney–Rivlin tests: In Figure 6, it is shown that the samples containing 5% resin, with both silanes, have a higher CLD than the reference compound. This is more evident in the equilibrium swelling results. In samples containing amino and epoxy silanes, the crosslinks are formed through the vulcanization process via the sulfur addition, but all the additional interactions facilitated by the new coupling system (hydrogen bonds, π–π stacking, or covalent bonding) are also present. These new interactions could contribute to the calculated CLD by swelling, enhancing the overall network structure.

**Figure 7:**
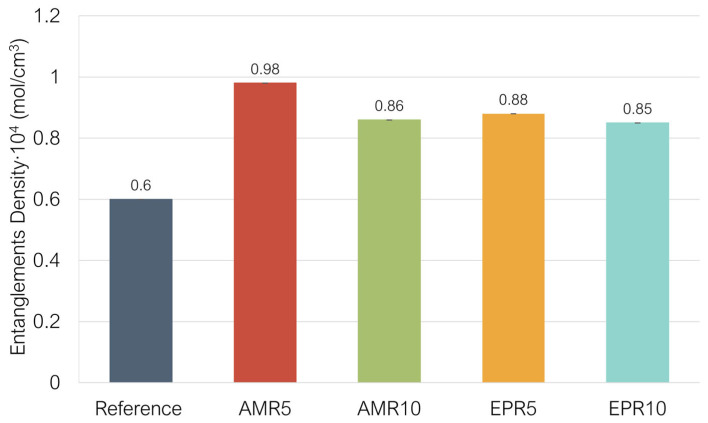
Entanglements density measured by the Mooney–Rivlin method applied to the studied compounds.

Furthermore, if the resin has good compatibility with the SBR, some physical interactions can be formed. The resin can be entangled in between the polymer chains of the SBR. All these additional interactions can contribute to a higher value of CLD measured by swelling in these compounds compared to the reference sample with TESPD. However, in the case of the CLD results obtained using the Mooney–Rivlin method, samples containing 5% resin exhibit a CLD comparable to that of the reference compound. This discrepancy with the swelling test results may arise from the fact that the Mooney–Rivlin method distinguishes between chemical crosslinks and physical entanglements. As such, the weaker additional interactions in the modified compounds are more likely to contribute to entanglement density rather than to measurable crosslink density. This is supported by the results in Figure 7, where all modified samples show higher entanglement densities than the reference, with AMR5 displaying the highest value.

When 10% of the resin is added, a slight decrease in the CLD is observed for the compounds containing both silanes. As mentioned in the vulcanization analysis, it could be that 5% of resin is an optimum amount of the material, and 10% is an excessive quantity. The increasing resin content can elevate the possibility that the phenolic resin interacts with the accelerator, deactivating it during the vulcanization process and leading to lower values of CLD. These results, combined with the ones obtained for the Payne effect, seem to indicate that the higher values of the Payne effect and the maximum torques observed in the curing curves are a combination of the CLD, filler–filler, and polymer–filler interactions.

### 3.5. Stress–Strain Behavior

**Figure 8:**
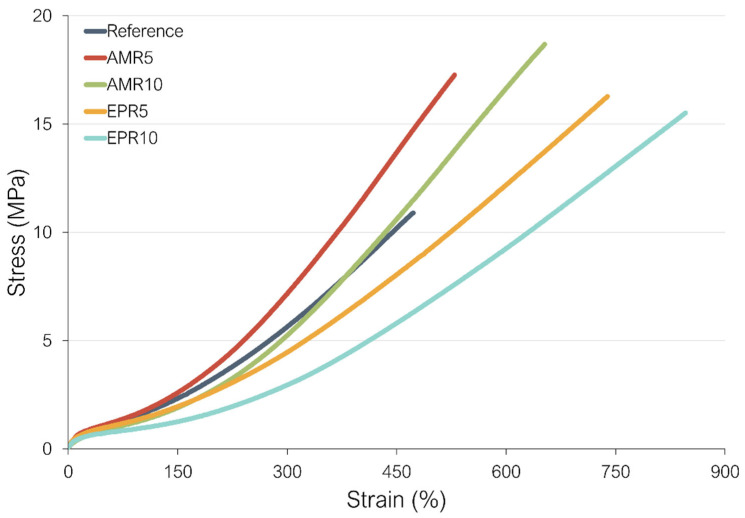
Stress–strain curves at RT of the rubber compounds.

The mechanical properties of the compounds studied in this work are shown in Figure 8 and Table 4. As can be seen, all samples including resin have a higher tensile strength (T_s_) and elongation at break (ɛ_b_) than the reference. The notable increase in elongation at break compared to the reference can be attributed to the slippage of entangled polymer chains connected to silica, facilitated by the additional interactions in the new system among silica, silanes, resin, and elastomer [18]. These increased interactions in the newly developed systems contribute to the higher tensile strength observed in these samples.

The samples containing amino silane also present a higher reinforcement index (ratio between the modulus at 300% strain and the modulus at 100% strain). Both samples with amino silane exhibit an improved mechanical performance compared to the sample with TESPD and to the samples with epoxy silane. The sample with amino silane and 5% of resin (AMR5) shows the best performance of all compounds. These results are in agreement with the ones obtained for the Payne effect, in which this sample had the highest G’ at high strains, indicating higher filler–rubber interactions in this compound. As observed already in the cure behavior, the Payne effect, and CLD, an increase in the amount of resin to 10% seems to have a negative effect on some in-rubber properties. Independently of silane used (amino or epoxy silane), the addition of 10% of resin caused a deterioration of the moduli compared to the samples with 5%. This result is another hint that 10% of resin is above the optimum amount that should be added into the rubber compounds. As previously mentioned, at this higher material concentration, the resin appears to deactivate the accelerator, resulting in a significantly lower CLD compared to the reference as well as the compounds containing 5% resin.

### 3.6. Dynamic Properties

The impact of the implementation of the new coupling on the dynamic properties of the rubber compounds was studied through the analysis of the loss factor (tan δ) as a function of temperature. The loss factor is defined as the ratio between the loss and the storage moduli, and it is employed as an indicator of tire performance. Usually, the values of tan δ at low (0 °C) and high temperatures (60 °C) are used as indicators of the wet grip (WG) and the rolling resistance (RR), respectively [47]. These two properties are key in tire performance and form—together with the abrasion resistance—the Magic Triangle of tires. However, the prediction for the RR is more accurate than the one of the WG. The reason for this is that the wet grip is a complex phenomenon that takes place within a high-frequency range of 10^4^ to 10^7^ Hz, and these frequencies are too high to be measured using DMA [47].

**Figure 9:**
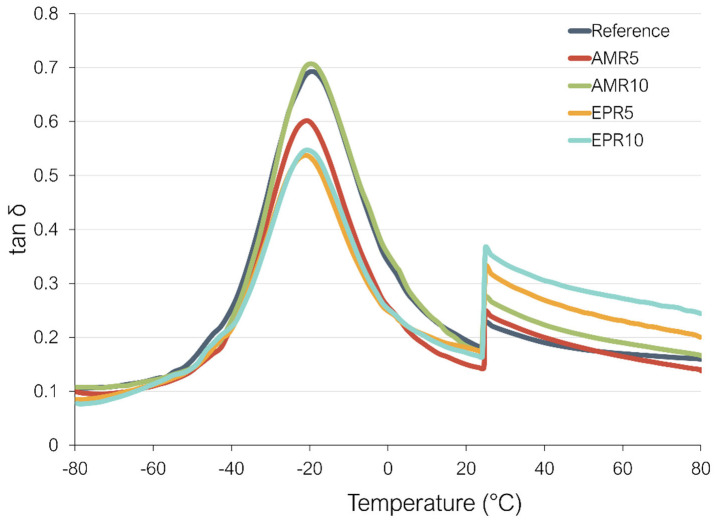
Variation in the loss factor (tan δ) as a function of temperature of the studied compounds (the sharp drop in the lost factor at 25 °C is caused by the increase in applied strain).

The variation in the loss factor as a function of temperature is shown in Figure 9.

As can be seen in Figure 9, the application of the new coupling system has a significant impact on the dynamical properties of the compounds. Substantial differences can be observed at both low and high temperatures. Observing the values of the loss factor at 0 degrees, commonly used as the wet grip indicator, it is shown that samples AMR5, EPR5 and EPR10, present a lower value than the reference. While sample AMR10, shows a slightly higher loss factor at 0 degrees. The lower loss factor for the samples AMR5, EPR5, and EPR10 could be caused by a stronger network in these compounds. As discussed before, it is assumed that these samples contain more interactions in the system than the reference, leading to a more elastic material and therefore to lower hysteresis. These samples contain not only the crosslinks formed during the vulcanization but also additional non-covalent interactions between silica, the resin, and silanes. Regarding the sample AMR10, with 10% resin, it could be, as previously discussed, that the optimum amount of resin is exceeded and that the resin is also acting as a plasticizer, showing a result more similar to the reference. This behavior is not observed in the case of the sample with 10% of resin and epoxy silane, because as discussed beforehand, the epoxy silane has a higher reactivity towards the resin due to the ring opening. This could lead to the presence of stronger (covalent) interactions compared to the sample with amino silane. However, it is known [48] that the prediction of the wet grip using this method is not as reliable as for the rolling resistance when two different systems are compared. Therefore, only a real tire test can show the real grip performance.

Regarding the rolling resistance indicator (tan δ) at 60 °C, some differences to the reference compound are observed. In this case, the behavior of the new coupling is extremely influenced by the type of silane used. The samples AMR5 and AMR10 with amino silane show a significantly lower RR indicator than the samples with epoxy silane. Samples containing amino silane exhibit a behavior which is more similar to the reference, with AMR5 displaying the lowest RR indicator among all compounds. This sample shows a CLD similar to the reference and exhibits stronger rubber–filler interactions, as evidenced by the highest G’ value at high strains in the Payne effect measurement. These interactions contribute to greater elasticity and, consequently, reduced energy dissipation. In the case of samples with epoxy silane, it could be that the higher energy dissipation at higher energy is caused by the bonding and debonding of the different interactions present in the system. This result could be a hint that the amount of these non-covalent interactions is higher in the compounds with epoxy silane.

Figure 9 also shows that the resin has good compatibility with the rubber matrix. When a non-compatible resin is used, a second peak in the tan δ curve is typically observed [49,50,51,52]. Therefore, in this case, without having such a second peak, the resin presents a good compatibility with the SBR.

### 3.7. Re-Connectivity of Bonds

After the analysis of the effects of the new coupling system on the in-rubber properties, the best-performing compound was selected for further analysis of its reversible nature and a recyclability study. The chosen compound was AMR5, in which 3-Aminopropyltriethoxysilane was used as silane together with 5% resin. The selection of this compound was performed because it showed the best overall in-rubber performance from all the newly designed compounds tested.

- Study of the mechanical response of the compounds at high temperatures

**Figure 10:**
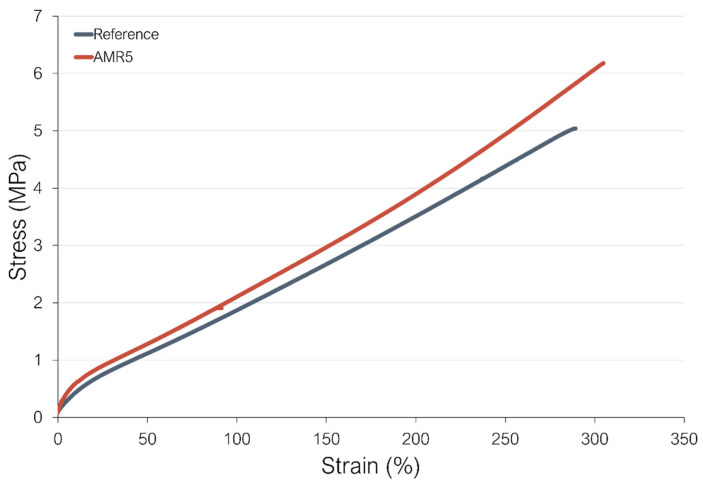
Stress–strain curves at 100 °C of the reference and AMR5.

The behavior of the new coupling at high temperatures was analyzed by testing the mechanical response of the compounds at 100 °C. The mechanical properties of the reference and the sample AMR5 are shown in Figure 10.

As observed in Figure 10, the sample AMR5 shows higher moduli at 100 and 300% strain, tensile strength, and elongation at break at 100 °C than the reference. This improved mechanical performance at elevated temperatures could be caused by the reversible nature of the non-covalent interactions present in this system. When a connection is broken in the compound AMR5 due to the applied strain, it is possible to re-connect. This leads to observed higher resistance to applied strain at elevated temperatures than in the reference compound with TESPD.

- Creep experiments

**Figure 11:**
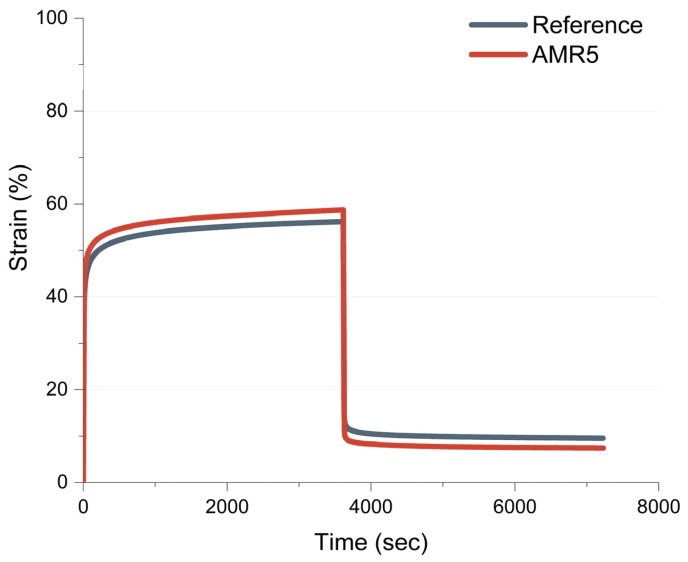
Variation in the strain during the creep experiments for the reference and AMR5.

In Figure 11, the results of the creep experiment at 100 °C are depicted.

When the initial force of 15 N is applied, the sample AMR5 reaches a slightly higher initial strain than the reference compound, approximately 50% for AMR5 and 47% for the reference. The stress is then maintained for one hour, in which the strain increases slowly until the stress is released. At this point, the sample AMR5 reaches a maximum strain of ~59% and the reference ~55%. Subsequently, when the stress is released, the compounds go back to their original shape. Both compounds almost regained their previous form. However, it can be observed that sample AMR5 shows a slightly higher recovery than the reference. This could indicate that the non-covalent interactions present in the new system provide the compound with a slightly higher thermo-mechanical fatigue resistance.

- Analysis of the changes in the dynamical properties after subjecting the vulcanizates to a cycling (fatigue) test

**Figure 12:**
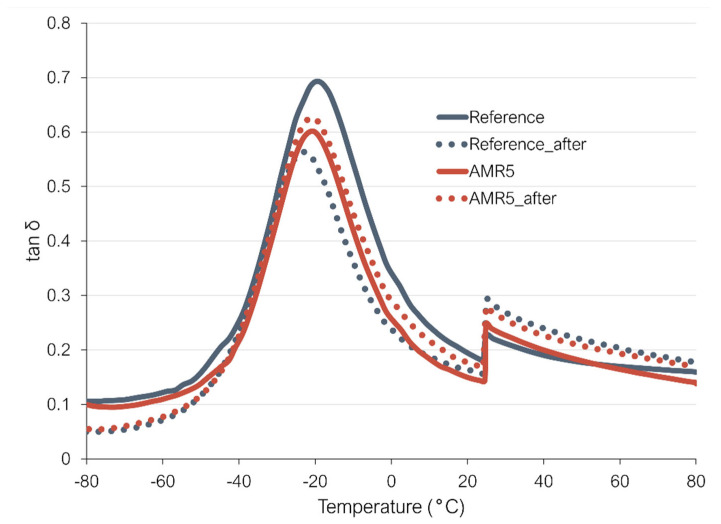
Variation in the loss factor as a function of temperature before and after the thermo-mechanical treatment (the sharp drop in the loss factor at 25 °C is caused by the increase in applied strain).

The dynamic properties of the selected compounds were analyzed before and after a fatigue test. Compound AMR5 and the reference were subjected to five tensile cycles, each stretched to 100% strain at 100 °C. Following this procedure, the dynamic properties were measured and compared with the values recorded before the cycling test. Figure 12 shows the evolution of the loss factor with temperature. Table 5 depicts the values of tan δ at 0 and 60 degrees.

As depicted in Figure 12, the variations between the curves before and after fatigue testing are less pronounced for the AMR5 sample compared to the reference compound. For the reference compound, the rolling resistance (RR) indicator rose by approximately 20%, while the wet grip (WG) indicator dropped by around 30%. In comparison, the AMR5 sample showed a 17% increase in the RR indicator and a 12% rise in the WG indicator. The compound with the new coupling shows better wet grip and rolling resistance indicators after the cycling test than the reference with TESPD. This suggests that the new coupling offers better resistance to higher temperatures and fatigue than the conventional silica/silane coupling.

### 3.8. Recycling Study

**Figure 13:**
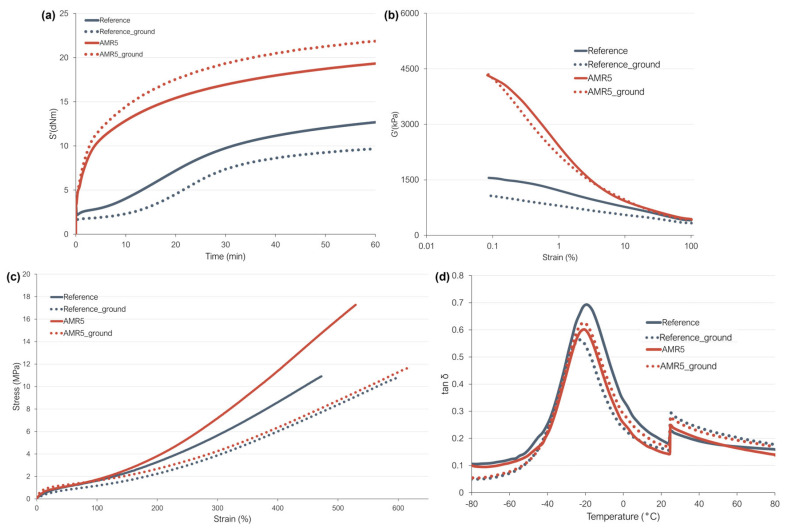
(**a**) Vulcanization curves, (**b**) Payne effect plots, (**c**) stress–strain curves and (**d**) variation in the loss factor as a function of temperature of the compounds analyzed in the recyclability study.

One of the main goals of the development of a new coupling based on non-covalent interactions is to facilitate the recyclability of silica-filled rubber compounds. In order to explore the advantages of the new coupling regarding recyclability, a recycling study was conducted with samples AMR5 and the reference. The primary objective of this test is to determine whether the introduction of a reversible coupling positively influences the recyclability of the rubber compounds. The samples were subjected to a mechanical treatment in which they were ground to smooth its incorporation into virgin rubber compounds as explained in the experimental section of this manuscript. Figure 13 depicts the curing behavior, the Payne effect, stress/strain curves, and the loss factor as a function of temperature of the compounds containing ground rubber material in comparison with the original compounds. In both cases, the ground particles are included in a compound of the same composition as the ground rubber, e.g., AMR5 ground particles were included in the virgin AMR5 compound. As can be seen, all the compounds containing ground rubber show a deterioration in the in-rubber properties. Such behavior was reported in previous studies [6,7,10].

Figure 13a presents the cure curves for the compounds. AMR5_ground exhibits a higher maximum torque than AMR5, while the opposite is true for the reference compound, where the compound with ground rubber shows a decrease in this parameter. As discussed before, the maximum torque can be related to the CLD, filler–filler and polymer–filler interactions. In the case of AMR5_ground, the increase in M_H_ could be caused by a combination of both, a higher CLD and an increase in all the interactions present in this system. For the reference compound, most likely the decrease in the maximum torque is caused by a decrease in the CLD. In a previous study [52], it was reported that the presence of ground rubber particles causes diffusion of sulfur towards these ground rubber particles. This phenomenon leads to an inhomogeneous distribution of the sulfur in the rubber samples and therefore an inhomogeneous CLD. This behavior could be less pronounced in the samples with the new coupling because the penetration of the sulfur is more difficult than in the reference compound. This can be explained by a higher overall network (crosslinks, filler–filler, and polymer filler interactions) in these samples, due to the possible reformation of the non-covalent interactions after breaking them during the grinding process.

In Figure 13b, the Payne effect curves are depicted. Sample AMR5 with ground material shows a similar Payne effect compared to its original counterpart. However, as previously considered, it is also possible that the Payne effect in these samples is a combination of both the breakdown of the filler network and the break of the non-covalent bonds present in the system. It might be possible that in sample AMR5_ground also the non-covalent interactions present are breaking with the applied strain. In the case of the reference, a significant decrease in the Payne effect is observed. This phenomenon can be attributed to the reduced quantity of fillers in these compounds. In the sample containing ground material, 15 phr of the silica filler was replaced by ground rubber. Since this material consists of rubber, fillers, curatives, and other components, the overall filler content in the compounds that include ground rubber is lower than in the original compound. This reduction results in decreased particle–particle interactions, which in turn leads to a reduced Payne effect.

The mechanical performance of the samples is shown in Figure 13c and Table 6. It can be seen that the compounds with ground material show a significant worsening of the mechanical properties. As explained before, this can also be caused by the inhomogeneity in CLD caused by the sulfur diffusion to the ground particles [53]. This behavior occurs in both the reference and the sample with the new coupling system. However, the deterioration of the mechanical performance is more pronounced in the sample with the new coupling system. The higher degree of deterioration of the properties for the sample AMR5_ground could be explained in addition by a worse micro-dispersion in this sample as observed by the Payne effect results.

Lastly, the dynamical mechanical properties of the compounds were analyzed. The loss factor as a function of temperature of the studied samples is shown in Figure 13d. As with the other properties, significant changes can be observed in the samples with ground material. However, in the case of the sample AMR5, the changes in the tan δ curve are smaller than in the reference compound. As mentioned before, the changes in the dynamical properties can be explained by the diffusion of sulfur to the ground particles [53] that is less prominent for the samples with the new coupling.

## 4. Conclusions

This study explores the development of a new silica/rubber coupling mechanism based on non-covalent interactions. The silica surface was modified with phenolic resin, which was grafted onto silica by reacting with silane that had previously interacted with the silica surface. Two different silanes were analyzed, 3-Aminopropyltriethoxysilane and 3-Glycidyloxypropyltrimethoxysilane. The modification of silica was performed during the mixing of the rubber compounds. The in-rubber results of the compounds containing the new coupling were compared to a conventional silica/silane system with TESPD.

This work revealed that the implementation of this novel coupling based on non-covalent interactions using amino silane and 5% of resin (sample AMR5) leads to an improvement in crosslink density, mechanical performance, and rolling resistance indicator. Tensile test results showed that the samples containing amino silane exhibit superior modulus, tensile strength, and elongation at break compared to the reference, indicating more efficient stress transfer between the filler and rubber matrix, likely due to enhanced filler dispersion and stronger interfacial interactions. Furthermore, these samples also show a promising dynamic behavior, with similar energy dissipation at 60 degrees (rolling resistance indicator) than the reference. However, the use of the new coupling has a negative effect on the Payne effect. Nevertheless, it is possible that the breaking and re-connection of the non-covalent interactions present in these systems is contributing to the higher values of the Payne effect. The improvement in the properties of the new compounds can be explained by the presence of a high number of additional interactions and a good compatibility of the phenolic resin with the rubber matrix. The new coupling also exhibits a reversible nature, which results in better performance when tested at high temperatures and after being subjected to mechanical fatigue. The creep testing supported this theory, showing thar under prolonged loading the new compound had reduced permanent deformation. Furthermore, the new coupling also shows encouraging results in the recyclability study, with a smaller deterioration of the properties compared to the virgin material.

This work shows the capability of the use of non-covalent interactions to develop a new silica/rubber coupling. This system has the potential to be optimized to obtain a higher improvement in rubber performance and recyclability.

## Figures and Tables

**Table 2:** Mixing procedure of rubber compounds.

Time (min)	Action
Stage 1. Pre-heating 100 °C—rotor speed 50 rpm	
0.00	Addition of rubber
1.20	Addition of 1/3 filler, 1/2 silane
2.40	Addition of 1/3 filler, 1/2 silane, TDAE
4.00	Addition of 1/3 filler and resin
5.00	Increase in temperature
10.00	End of mixing (reaching 140 °C)
Stage 2. Pre-heating 100 °C—rotor speed 50 rpm	
0.00	Addition of batch from stage 1
1.00	Addition of ZnO and Stearic Acid
1.20	Increase in temperature
5.00	End of mixing (reaching 140 °C)
Stage 3. Pre-heating 50 °C—rotor speed 50 rpm	
0.00	Addition of batch from stage 2
1.30	Addition of curatives (Sulfur, TBBS)
3.00	End of mixing

**Table 3:** Payne effect (ΔG′) and G′ at 100% strain of the studied compounds.

Compound	ΔG′ (kPa)	G′ at 100% Strain (kPa)
Reference	1150	400
AMR5	4340	530
AMR10	3700	420
EPR5	3880	440
EPR10	4010	370

**Table 4:** Mechanical properties of rubber compounds at RT.

Compound	Reinforcement Index (M_300%_/M_100%_)	T_s_ (MPa)	ɛ_b_ (%)
Reference	3.6 ± 0.01	10.7 ± 1.8	470 ± 60
AMR5	3.6 ± 0.1	17.5 ± 0.7	600 ± 80
AMR10	3.9 ± 0.1	19.0 ± 1.2	680 ± 70
EPR5	2.8 ± 0.5	15.7 ± 1.9	780 ± 90
EPR10	3.0 ± 0.2	16.6 ± 1.1	880 ± 70

**Table 5:** Values of tan δ at 60 °C and 0 °C of the studied compounds before and after the thermo-mechanical treatment.

Compound	tan δ at 60 °C	tan δ at 60 °C After Cycling 5 Times at 100 °C	tan δ at 0 °C	tan δ at 0 °C After Cycling 5 Times at 100 °C
Reference	0.169	0.203	0.352	0.244
AMR5	0.164	0.192	0.266	0.299

**Table 6:** Mechanical properties of the compounds analyzed in the devulcanization study.

Compound	Reinforcement Index (M_300%_/M_100%_)	T_s_ (MPa)	ɛ_b_ (%)
Reference	3.6 ± 0.12	11.6 ± 1.8	470 ± 60
Reference_ground	3.2 ± 0.12	10.7 ± 1.0	630 ±30
AMR5	3.6 ± 0.1	17.5 ± 0.7	600 ± 80
AMR5_ground	3.1 ± 0.2	9.5 ± 0.3	620 ± 40

## Data Availability

Data are contained within the article.
